# Policy approaches to health system performance assessment

**DOI:** 10.2471/BLT.24.292104

**Published:** 2024-07-01

**Authors:** Irene Papanicolas, Dheepa Rajan, Marina Karanikolos, Dimitra Panteli, Kira Koch, Faraz Khalid, Gerard Schmets, Suraya Dalil, Josep Figueras

**Affiliations:** a Center for Health System Sustainability, Brown University, Providence, United States of America.; b European Observatory on Health Systems and Policies, World Health Organization European Centre for Health Policy, 40 Place Victor Horta, 1060 Brussels, Belgium.; c Special Programme on Primary Health Care, World Health Organization, Geneva, Switzerland.

Improving health system performance is a priority for policy-makers. As the population ages and the burden of chronic disease grows, governments are spending more on health-care provision.^1^ Unpredictable and catastrophic threats such as pandemics, extreme weather events and sociopolitical crises require resilient and adaptable health systems. Therefore, policy-makers need reliable and timely information to identify the strengths and weakness of health systems and a broad evidence base to help them shape policy approaches to achieve health system goals.

Health system performance assessment is a comprehensive evaluation process designed to measure how well a health system achieves its objectives and identify opportunities for improvement. The multifaceted nature of health systems, the diversity of data and stakeholders involved, and the dynamic and context-dependent environment in which health systems operate make these assessments a complex endeavour. While efforts to embed health system performance assessment in decision-making have recently intensified, innovative approaches that transform evidence into actionable policies are still needed.

Assessing performance requires identifying all components of a health system – and its boundaries. A clear framework for health system performance assessment can help identify which elements within the health system are important to measure, how these are linked to the system’s ability to deliver objectives and what broader factors may affect performance.^2^ In this theme issue, a perspective outlines the progress made in collecting information on the structure and functions of health systems, noting the gaps and arguing for more harmonization of information collected across health system performance assessment tools.^3^ Another reveals the intersections with other sectors and advocates for a holistic view of these assessments, emphasizing that achieving health system goals can enhance overall societal well-being.^4^ To understand the effects of multisectoral interventions on health system performance, an article explores available literature.^5^ The specific challenges in using a health systems performance assessment framework to assess public health systems are also considered,^6^ as well as how to adapt these frameworks to country contexts and policy cycles.^7^

A robust health system performance assessment is inextricably linked to data availability and quality. While measures of health inputs such as number of facilities may be broadly available across countries, quality metrics and patient-reported outcomes are not. A study reviews the indicator availability for primary care monitoring across five South Asian countries, highlighting existing data pockets and gaps, as well as issues with timeliness and harmonization.^8^ Another examines the use of DHIS2 – a web-based health management information system platform used in over 80 countries – for health service evaluation in three regions in Ethiopia,^9^ highlighting its potential for health system performance assessment at subnational level. A research study presents data from the People’s Voice Survey to compare utilization, experience and confidence in health systems across 16 countries,^10^ exploring how people’s perspectives can inform health system performance assessments. The use of the Hospital Consumer Assessment of Health Providers and Systems survey for performance assessment in Odisha, India is explored in another article.^11^ The paper finds that the factors influencing personal experience may vary even when the same tool is used, suggesting a need for caution in comparing such metrics across countries and even population groups within countries.

Health systems performance assessments can provide evidence that can inform policy, as explored by one article presenting a country-level assessment from Oman.^7^ The complexities of health systems have led researchers to use analytic approaches to evaluate policies. An article outlines different methods that can be used, as well as promising new data sources, by leveraging digital technologies and big data for data collection and analysis.^12^ Health system comparisons are often used as a tool for drawing insights on the relative performance of health systems; one of the perspectives^13^ urges researchers to make better use of existing information on health system characteristics to identify the appropriate cross-country comparators for the questions being asked. Finally, an article^14^ provides a useful illustration of cross-country analysis that can examine and compare health system resilience.

The articles in this issue highlight the importance of regular health systems performance assessment to inform policies that advance progress on health system objectives globally, and offer insights on associated data, methods and applications.
